# Cardiovascular Involvement in Tuberculosis: From Pathophysiology to Diagnosis and Complications—A Narrative Review

**DOI:** 10.3390/diagnostics13030432

**Published:** 2023-01-25

**Authors:** Dragos Traian Marius Marcu, Cristina Andreea Adam, Florin Mitu, Carmen Cumpat, Viviana Aursulesei Onofrei, Mihai Lucian Zabara, Alexandru Burlacu, Radu Crisan Dabija

**Affiliations:** 1 Department of Medical Specialties (I and III) and Surgical Specialties, Grigore T. Popa University of Medicine and Pharmacy, University Street No. 16, 700115 Iaşi, Romania; 2 Clinical Hospital of Pneumophthisiology Iași, Doctor Iosif Cihac Street no 30, 700115 Iasi, Romania; 3 Clinical Rehabilitation Hospital, Cardiovascular Rehabilitation Clinic, Pantelimon Halipa Street No. 14, 700661 Iasi, Romania; 4 Academy of Medical Sciences, Ion C. Brătianu Boulevard No 1, 030167 Bucharest, Romania; 5 Academy of Romanian Scientists, Dimitrie Mangeron Boulevard No. 433, 700050 Iasi, Romania; 6 Department of Management, Alexandru Ioan Cuza University, Carol I Boulevard, 700506 Iasi, Romania; 7 St. Spiridon Clinical Emergency Hospital, Independence Boulevard No. 1, 700111 Iasi, Romania; 8 Institute of Cardiovascular Diseases George I.M. Georgescu, 700503 Iasi, Romania

**Keywords:** tuberculosis, cardiovascular involvement, pericarditis, myocarditis, aortitis, cardiotoxicity

## Abstract

Although primarily a lung disease, extra-pulmonary tuberculosis (TB) can affect any organ or system. Of these, cardiovascular complications associated with disease or drug toxicity significantly worsen the prognosis. Approximately 60% of patients with TB have a cardiovascular disease, the most common associated pathological entities being pericarditis, myocarditis, and coronary artery disease. We searched the electronic databases PubMed, MEDLINE, and EMBASE for studies that evaluated the impact of TB on the cardiovascular system, from pathophysiological mechanisms to clinical and paraclinical diagnosis of cardiovascular involvement as well as the management of cardiotoxicity associated with antituberculosis medication. The occurrence of pericarditis in all its forms and the possibility of developing constrictive pericarditis, the association of concomitant myocarditis with severe systolic dysfunction and complication with acute heart failure phenomena, and the long-term development of aortic aneurysms with risk of complications, as well as drug-induced toxicity, pose complex additional problems in the management of patients with TB. In the era of multidisciplinarity and polymedication, evidence-based medicine provides various tools that facilitate an integrative management that allows early diagnosis and treatment of cardiac pathologies associated with TB.

## 1. *Mycobacterium tuberculosis*—An Old but “Still Standing” Enemy

Tuberculosis (TB) is a public health problem that, despite sustained global efforts, claimed 1.3 million deaths globally in 2020, with an upward trend to 1.4 million people in 2021. Difficulties in diagnosing and treating TB cases during the COVID-19 pandemic reversed years of progress in the field [1,2]. According to the WHO 2022 Incidence Report, about 10.6 million people became ill with TB in 2021, with immunocompromised patients accounting for about 7% of the total [3]. Epidemiological data published for the year 2021 show a higher infection rate among men compared to women [4]. Historical records refer to genus *Mycobacterium* as having existed over 150 million years ago [5]. Mentions of this infection are also found in the Old Testament, when Egypt was known as a geographical area with an extremely high prevalence of this pathology referred to at that time as ʺconsumptionʺ (from the Latin *consumptio*) [6,7,8,9]. Responsible for the appearance of severe pulmonary and extrapulmonary infections, *Mycobacterium tuberculosis* (MTB) has over time been attributed various names such as "*schachepheth*” [10], “*phthisis*”, or “*king’s evil*’ [11,12]

Although primarily a lung disease, extra-pulmonary TB can affect any organ or system. Of these, cardiovascular complications associated with disease or drug toxicity significantly worsen the prognosis [13]. Cardiovascular involvement primarily affects the pericardium, and only in very rare cases the myocardium or other cardiac structures. TB-associated pericarditis occurs frequently in immunocompromised patients associated with HIV infection, and less frequently in immunocompetent patients. In rare cases, whether associated with pericarditis or not, left ventricular systolic dysfunction occurs; the symptoms of heart failure may be overlooked as they overlap the general symptoms of TB [14,15].

This article aims to review the latest information from the literature on the diagnosis and management of cardiac involvement in TB based on the need to implement simple diagnostic algorithms that allow prompt initiation of TB treatment and monitoring of potential cardiotoxic effects associated with it.

## 2. Materials and Methods

We searched the electronic databases PubMed, MEDLINE, and EMBASE for studies that evaluated the impact of TB infection on the heart (including research from 1956 to present), with the aim of conducting a review of recent literature on the impact of infection on the cardiovascular system from pathophysiological mechanisms to clinical and paraclinical diagnosis of extrapulmonary damage as well as the cardiotoxicity associated with antituberculosis medication.

We used the following words or phrases for our search: ʺ*Mycobacterium tuberculosis*ʺ plus one of the following (in various associations)—ʺheartʺ, ʺcardiac involvementʺ, ʺpericarditisʺ, ʺmyocarditisʺ, ʺcoronary artery diseaseʺ, ʺaortitisʺ, ʺintracardiac tuberculomaʺ, ʺantituberculosis medicationʺ, and ʺcardiotoxic effectʺ. Observational studies, including prospective or retrospective cohort studies, RCTs, meta-analyses, guidelines, and case reports related to our topic were included. We also manually searched the reference sections of the identified articles for additional publications. Two independent reviewers selected studies by analyzing the title and abstract.

## 3. Tuberculosis—Much More than a Pulmonary Disease

Extrapulmonary TB can affect any organ, making diagnosis difficult without high serum bacterial titers [16,17,18]. The most common sites are the pleura and lymph nodes, but in rarer cases the bones, joints, peritoneum, kidneys, or meninges may also be affected. The therapeutic management of these forms can be challenging for clinicians, as these patients often require treatment over a longer period of time, with monitoring for potential adverse effects, toxicity of therapeutic agents, or complications associated with affected organs.

Adherence to treatment remains one of the main problems, being one of the main reasons for lack of therapeutic efficacy [18,19]. Cardiovascular involvement is not to be neglected but the diagnosis is often difficult. Pottenger [20] first described the presence of heart disease secondary to MTB infection in the early 1900s. Advances in technique and refinement of diagnostic and treatment algorithms now allow fairly easy identification of extrapulmonary TB-associated disease, and it is now estimated that about 60% of patients with MTB have consecutive cardiac damage [21,22,23]. In addition to the traditional cardiovascular risk factors of high blood pressure, diabetes mellitus, dyslipidemia, and obesity, clinical studies in the literature denote MTB as a promoter of atherosclerosis via its pronounced inflammatory effect, thus indirectly contributing to the development and progression of cardiovascular diseases [24,25,26,27]. The pathophysiological substrate by which MTB is associated with the occurrence and progression of atherosclerotic lesions is the associated pro-inflammatory effect from the endothelial cell wall [28,29,30]. The differential diagnosis of TB (pulmonary or extrapulmonary) can in many cases be challenging because of the variable clinical picture. TB and sarcoidosis have in many cases a similar clinical, immunological, and radiological presentation, which raises difficulties in establishing a positive diagnosis [31,32]. Morpho-pathologically, TB is a caseous granulomatous disease, whereas sarcoidosis is characterized by the presence of non-caseous epithelioid granulomas [33].

At the moment, it is recommended that recognizing cardiovascular involvement in tuberculosis follow a complex diagnosis, with dosing of ADA and interferon-γ, as well as advanced imaging techniques such as CT and MRI [34].

## 4. Cardiovascular Involvement in TB

**Figure 1:**
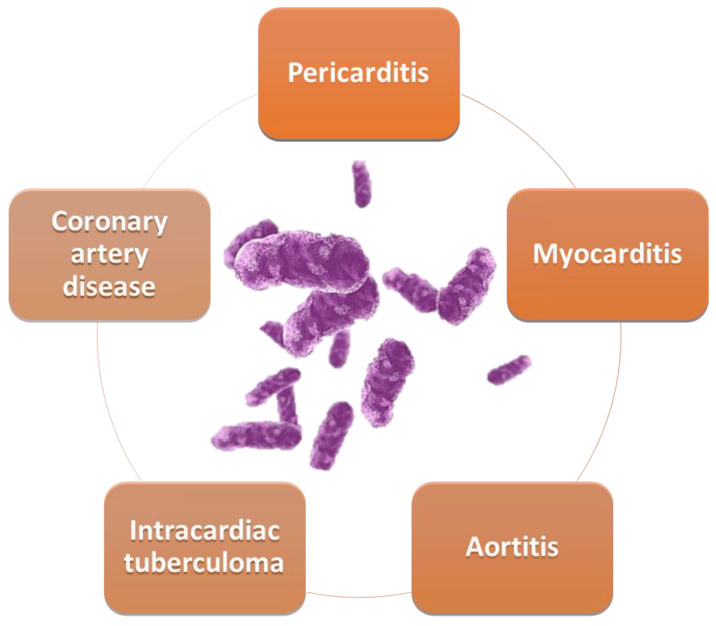
Cardiovascular involvement in TB.

The cardiovascular structures most involved in tuberculosis are the pericardium, myocardium, and aorta (Figure 1).

### 4.1. Tuberculous Myocarditis

Myocardial involvement may be associated with pericarditis in the form of myopericarditis, or it may cover other clinical scenarios. Tuberculous myocarditis is particularly rare, with an estimated prevalence of less than 2% [34,35].

The first two cases of myocardial damage in TB were reported in 1664 by Maurocadat and in 1761 by Morgagni [36]. Epidemiological studies show a predominance of tuberculous myocarditis in patients under 45 years old, and it is twice as common in male patients [15]. Invasion of the myocardium by MTB is realized via the hematological route, by retrograde lymphatic insemination from the mediastinal nodes, or by direct invasion from the pericardium [37,38,39]. The different effects of MTB on the pericardium and myocardium can be explained on the one hand by the continuous movement of the myocardium, which indirectly prevents the lodging of bacilli, and on the other hand by the lactic acid produced, which has a protective role against bacilli [36,40].

Most cases have been reported in immunocompromised patients, who frequently have an HIV infection. Reported cases have frequently affected the left heart, and especially the left ventricle. Predominant right mediastinal lymph node involvement has been observed in many patients with MTB-induced myocarditis, which increases the risk of right heart damage by contiguity [41].

Myocardial damage is frequently asymptomatic, sometimes with severe consequences, leading to forms of acute heart failure [42,43]. In these cases, involvement of the right mediastinal lymph nodes has been observed, with a greater chance of contiguous involvement of the right side of the heart [15]. Identified forms include nodular myocardial damage with central caseation, miliary forms, or diffuse, inflammatory, giant cell forms [44].

A significant percentage of patients with myocardial damage also have pericarditis, which further worsens the prognosis [15]. Studies presenting data from endomyocardial biopsies indicate predominantly biventricular involvement in about 70% of cases, with isolated right ventricular dysfunction occurring in a small percentage of cases, only 8% [45].

The symptomatology of patients with myocarditis secondary to TB is variable, ranging from no symptoms to severe forms presenting at onset, with ventricular arrhythmias [46], sudden death, long QT syndrome [47], atrioventricular blocks, or clinical signs of congestive heart failure [42,48,49]. The clinical picture of these patients includes in some cases electrolyte imbalances, one of the most frequently reported being hypercalcemia [50].

The diagnostic criteria for myocarditis are the classic ones, represented by the identification of a high titer of myocardial enzymes together with the echocardiographic presence of left ventricular systolic dysfunction [15,37,51]. Nuclear magnetic resonance is an essential investigation in patients with myocarditis, highlighting in the T2 sequence a central and peripheral hypointense signal as well as a hyperintense thin line [52,53,54,55].

There are few data reported in the literature on the treatment of patients with tuberculous myocarditis, with clinical trials recommending the initiation of etiologic treatment. Improvement in symptoms does not eliminate the associated risk of sudden death, so these patients require regular monitoring, most often by multidisciplinary teams [56,57].

The most common complications reported were atrial fibrillation and sudden cardiac death [58]. Fulminant forms of MTB myocarditis can have an unfavorable outcome, especially in immunocompromised patients. Clinical studies report that 80% of fatal cases occur in female patients with associated LV systolic dysfunction [15].

Cases of chronic heart failure with preserved systolic function due to extensive intra-myocardial calcifications associated with latent MTB infection have also been reported [59].

### 4.2. Coronary Artery Disease and Tuberculosis

One interaction being considered is that between TB and coronary atherosclerosis, the presence of TB being associated with a 1.76-fold increased risk of developing coronary artery disease [27,60]. Implicitly, patients with TB have an associated risk of acute myocardial infarction of 1.98 compared to a similar cohort of patients without TB [23,61,62].

Both TB and ischemic coronary artery disease are common in developing countries and their association is all the more frequent. Several mechanisms are thought to be behind this. A first mechanism is related to a chronic inflammatory reaction, cell-mediated immune activation with the release of cytokines and chemokines, following latent infection. A second mechanism is the initiation of an autoimmune process following chronic infection, with production of antibodies against mycobacterial heat shock protein-65 (HSP65) [63]. This causes an induced cross-reaction with human HSP65, leading to endothelial injury and stimulating atherogenesis.

Heat shock proteins are a homogeneous group of proteins that arise in response to stress factors, originally discovered as a reaction to heat, hence the name. They show a high homogeneity between species and have in particular a chaperone role, but also mediate immune reactivity in certain diseases [64]. Animal model studies have shown that HSP65 inhibition affects IL-10 and paraoxonase-1 activity, while interferon-γ expression, myeloperoxidase activity, and the high-density lipoprotein inflammatory index tend to increase, leading to generalized as well as aortic atherosclerosis [65]. It has been observed that latent infection also results in elevated levels of interferon-γ, which may be a good predictor of progression to clinically manifest disease [66,67]. Moreover, considering that HSP65 mediates the early stages of the atherogenesis process, it is also being studied for the development of an anti-atherosclerotic vaccine [68]. A population-based study of more than 10,000 patients showed a 1.4-fold increased risk of acute coronary syndrome in patients diagnosed with TB compared to the general population [69]. This effect may be related to a combination of factors, with lung inflammation in general presumed to induce systemic inflammatory response, endothelial dysfunction, and atheroma plaque destabilization [70].

Recent data on latent TB have shown a high prevalence of ischemic coronary artery disease among these patients. Even in the absence of clinically manifest TB, chronic immune response to MBT can intensify the atherosclerotic process [15]. Another recent study showed a twofold increased likelihood of association of latent TB with acute myocardial infarction, after correcting for classical cardiovascular risk factors and other confounders [61]. Furthermore, it appears that vascular damage is not limited to the coronary arteries, and an association of latent TB with both peripheral arterial disease and ischemic stroke has been observed [71,72].

C-reactive protein (CRP), total white blood cell count, and neutrophil-to-lymphocyte ratio are three independent inflammatory predictors associated with a negative prognosis in coronary artery disease [73,74]. Serum CRP correlates with MTB bacterial load in sputum, having prognostic value and being associated with a high risk of death [75,76,77].

The beneficial effect of statins in reducing associated cardiovascular risk and decreasing systemic inflammation has been demonstrated, but its modulatory role in combination with MTB has not been fully elucidated to date [78,79,80,81,82]. There is a causal relationship between cholesterol and MTB, the bacterial agent needing cholesterol for infection and survival, with the caveat that the progression of infection is correlated with the ability of the immune system to limit infection [83,84]. Oxidized low-density lipoprotein plays an important role in patients with type 2 diabetes mellitus and TB, playing a central role in the formation of lipid-loaded foamy macrophages that contribute to the progression of tuberculous granulomas through lysosomal dysfunction [85,86,87].

Thus, various preclinical studies are reported in the literature in which statin administration is associated with stimulation of autophagy and phagosome maturation of MTB-infected macrophages. Research in murine models also highlights the beneficial role of statin administration in enhancing the therapeutic effect of first-line antituberculous drugs [88,89,90,91].

Statins have a beneficial role in the treatment of patients with TB infection and can be used as an adjuvant medication to standard treatment [83,92,93,94]. Administration of this hyperlipidemic medication increases cell resistance to MTB, but further clinical trials are needed in this research direction [92,95,96,97,98].

### 4.3. Tuberculous Pericarditis

Tuberculous etiology of pericarditis is one of the most common along with the neoplastic etiology [99], with a prevalence depending on a country’s level of development [100]. Approximately 1–2% of TB patients have associated pericarditis [101]. This type of pericarditis is characterized by a significant inflammatory status [102], chronicity [101], and a high risk of progression to a constrictive form [103].

Pericardial insemination with MTB occurs retrogradely, by the lymphatic route, by hematological dissemination, or in rare cases by direct damage to surrounding structures such as the lungs, pleura, or spine [104]. In the case of HIV co-infection, the pathway of dissemination is hematological [105]. The most common form of presentation of tuberculous pericarditis is the effusive form (in about 80% of cases), the constrictive form being considered one of the most common sequelae [106].

The prevalence of the constrictive form of pericarditis is reduced in patients without TB [100]. In patients infected with MTB, this form of the disease is seen in 25% of cases, but this percentage may be higher than for other forms such as idiopathic or viral [107].

**Figure 2:**
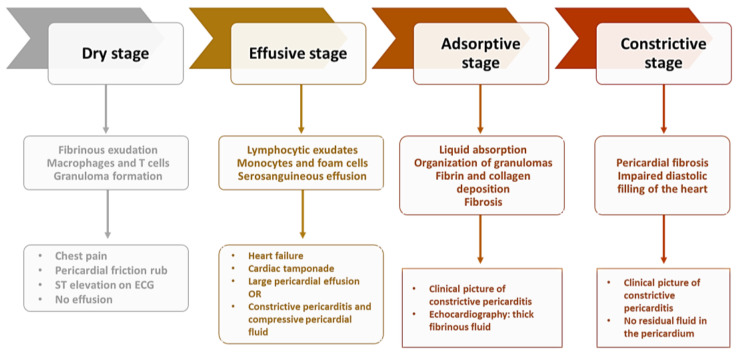
Stages of constrictive pericarditis (adapted after [105]).

Some patients have an atypical clinical form that poses problems of diagnosis and treatment, often delaying the latter and thus worsening the prognosis of patients. Tuberculous pericarditis presents four distinct stages, with a clinical picture and imaging features specific to pathophysiological processes (Figure 2) [13].

The dry stage is the least common, despite the marked symptoms that accompany it. The effusive stage is most often diagnosed by echocardiography, with a corresponding clinical worsening of the patient’s general status through the appearance of heart failure or even cardiac tamponade. The constrictive stage is encountered in a variable percentage of patients ranging from 5–25%, representing the stage with the most reserved prognosis in terms of associated dysfunction.

**Figure 3:**
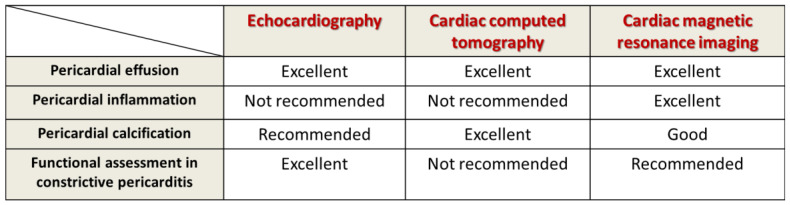
Multimodal imaging evaluation in tuberculous pericarditis (adapted after [111]).

Multimodal imaging evaluation of patients with pericardial effusion includes echocardiography, computed tomography, and nuclear magnetic resonance to differentiate the constrictive form from restrictive cardiomyopathy, which is the main entity with which the differential diagnosis is made [108,109,110] (Figure 3).

**Figure 4:**
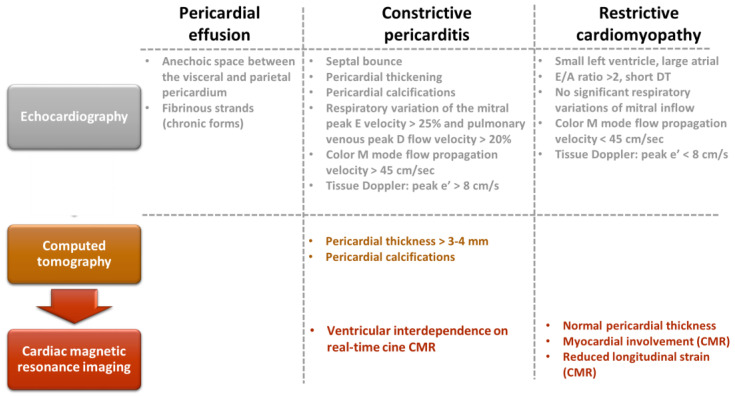
Multimodal imaging evaluation of patients with pericardial effusion, constrictive pericarditis, and restrictive cardiomyopathy (adapted after [114]).

Karima et al. [112] analyzed a group of 25 patients with constrictive pericarditis and demonstrated a high prevalence of infectious etiology as well as a statistically significant association with the presence of right ventricular dysfunction in this category of patients. Echocardiography, computed tomography, or nuclear magnetic resonance are the main imaging investigations used in the diagnostic algorithm of pericardial effusion [113]. The main findings identified in constrictive pericarditis as well as echocardiographic arguments establishing the differential diagnosis with restrictive cardiomyopathy are shown in Figure 4.

If transthoracic echocardiography provides suboptimal or inconclusive images (especially in the context of high suspicion of cardiac tamponade), transesophageal echocardiography is recommended. Cardiac CT is the gold standard in the evaluation of pericardial calcifications. Contrast-enhanced CT is recommended to avoid overestimation of pericardial effusion and to prevent artefacts from being missed on native examination. Nodular areas with increased attenuation, calcification of the anterior pericardium, and lack of changes when changing position in decubitus or in the presence of contrast enhancement of pericardium are arguments in favor of pericardial thickening [99,114,115].

Given the fact that a high percentage of patients have concomitant pulmonary and extra-pulmonary involvement, the management of these patients must be integrative, focused on the use of imaging methods. In addition to the above, chest ultrasound is another assessment method with applications in the management of patients with chest TB. Chest ultrasound allows detection of TB, dynamic follow-up of pleural effusions after evacuation, biopsy, or assessment of nodular involvement in children [116,117,118].

Geographical location often guides the diagnosis of a pericardial effusion, and there are a number of arguments for a tubercular etiology in endemic countries [119]. Thus, the identification of MTB in the stained smear or pericardial fluid culture and the presence of granulomas on histopathological examination confirm the diagnosis of tuberculous pericarditis [120]. The presence of pericarditis in a patient diagnosed with TB, an increased adenosine deaminase activity (ADA) activity and a high percentage of lymphocytes in the pericardial fluid, and a favorable clinical response secondary to the initiation of antituberculous treatment provide diagnostic clues, but further clinical tests are required to establish a positive diagnosis [106,115].

In some particular situations, obtaining negative serological tests does not exclude the tuberculous etiology of a pericardial effusion, sometimes requiring biopsy [16]. Patients with constrictive tuberculous pericardial disease have elevated levels of pericardial inflammatory cytokines such as IL-10 (*p* = 0.006) and interferon-gamma (*p* = 0.03). Ntsekhe et al. [121] analyzed a cohort of 91 patients with constrictive pericarditis, 68 of whom had TB, and using statistical regression analysis identified right atrial pressure above 15 mmHg (odds ratio of 48, *p* < 0.001) and serum IL-10 levels above 200 pg/ml (*p* = 0.04, 10 times higher associated risk) as predictors associated with calcification of the pericardium. In addition to inflammatory cytokine changes, anemia is one of the most common hematological changes seen in TB patients. The incidence reported in the literature varies, from 32% to 94%, most commonly in normochromic, normocytic forms [122,123].

De Vita et al. [124] reported the case of a 21-year-old patient with TB pericarditis as the first manifestation in whom pericardial fluid analysis was negative for MTB infection. Positive diagnosis for TB was established by positive urine lateral flow lipoarabinomannan assay, which required initiation of antituberculosis treatment. The glycolipid lipoarabinomannan is released during metabolism and degradation of infected cells in patients with active TB, leading to downregulation of pathophysiological processes that reduce interferon-gamma and interleukin-12 secretion [125,126]. Since its first use in 2001 to date, several clinical trials have been conducted to test the efficacy of this diagnostic test, but the reported results show low sensitivity [127,128].

The concurrent presence of HIV infection negatively modulates the prognosis of patients with tuberculous pericarditis, with an associated twofold increased risk of death [129]. In patients with tuberculous pericarditis without HIV infection, the virulence of the pathogen and the immune response secondary to interactions in the pericardium correlate with the course of the disease and thus with the patients’ medium- and long-term prognosis [130,131]. In HIV-positive patients, pericardial involvement frequently occurs through dissemination, in which case the infection plays the main prognostic role [105].

Mayosi et al. analyzed a cohort of 185 patients under observation with tuberculous pericarditis and observed that the mortality rate in this category of patients was higher in patients with clinical signs of HIV (*p* = 0.001) as well as in those with hemodynamic instability (odds ratio 1.80). Pericardiocentesis decreased this associated risk in the African cohort studied (odds ratio 0.34) [129]. Current European Society of Cardiology practice guidelines recommend pericardiocentesis under local anesthesia and ultrasound guidance for moderate and large pericardial effusions [115]. If prolonged drainage is required, it is recommended to opt for insertion of an indwelling catheter that will persist until drainage decreases to less than 20-30 ml per day [113]. In the case of patients with high risk associated with pericardiocentesis—those with recurrent or malignant forms—a safer pericardial window is recommended [132]. In many cases where there is a high suspicion of tuberculous pericarditis, in addition to pericardial fluid analysis, pericardial biopsy and histopathological analysis of the tissue fragment is recommended to identify pathognomonic granulomas [133].

**Figure 5:**
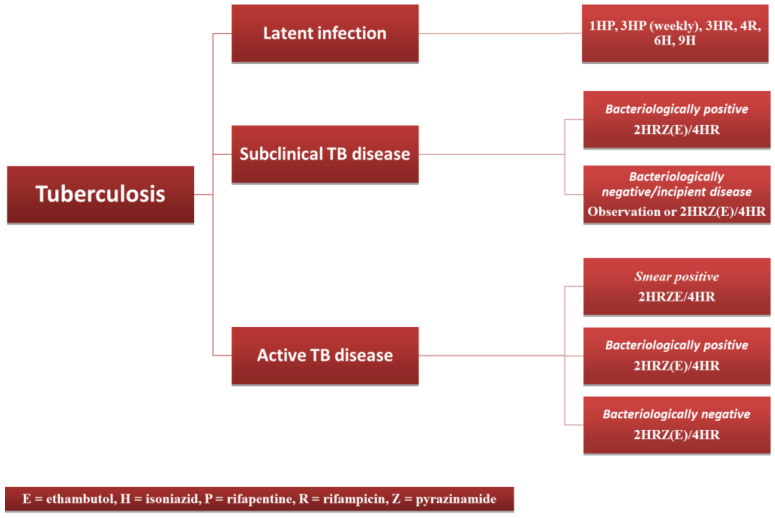
Therapeutic management of TB (adapted after [134]).

Antituberculous treatment is complex, based on combinations of therapeutic agents over long periods of time, which brings the risk of resistance to treatment. Currently, five main therapeutic agents are used: ethambutol, isoniazid, rifapentine, rifampicin and pyrazinamide. Depending on the type of associated infection (latent, subclinical, or active), several treatment regimens have been developed, which are shown in Figure 5. Patients with active TB undergo a 6-month multidrug treatment, which is divided into two distinct phases: an intensive 2 month phase, in which three to four antituberculous drugs are administered (isoniazid, rifampicin, pyrazinamide, and possibly ethambutol), and a continuation phase, in which dual therapy with isoniazid and rifampicin is administered for 4 months [134,135].

Surgical intervention is recommended in patients in whom constrictive pericarditis persists [136] or in the case of those without constriction in the context of lack of improvement of the clinical picture after 6–8 weeks of antituberculosis treatment [130]. Clinical studies to date have shown persistently high rates of morbidity and mortality in patients with tuberculous pericarditis despite specific therapy. Mayosi et al. [137] analyzed a cohort of 1400 adults with TB for the efficacy of consecutive administration of glucocorticoids and *Mycobacterium indicus pranii* immunotherapy. Administration of prednisolone induced a lower rate of occurrence of isolated constrictive pericarditis (*p* = 0.009) and a shorter duration of hospitalization (*p* = 0.04) compared to placebo, but had no statistically significant effect on the composite endpoint of mortality rate, occurrence of cardiac tamponade requiring pericardiocentesis, or constrictive pericarditis. In another recent clinical study, Steigler et al. [138] demonstrated that immunotherapy does not significantly contribute to the improvement of the associated pro-inflammatory status by modulating the mycobacteria-specific T cell response, thus supporting previous results. Pericardiectomy leads to improved death rates in TB patients and improves patients’ symptoms, with Yadav et al. showing improvement in NYHA class at one year [139]. Sclerosing therapy is also used in patients with recurrent pericardial effusions, predominantly of neoplastic etiology. It consists of intrapericardial administration of various therapeutic agents such as colchicine, bleomycin, cisplatin, or tetracyclines with the aim of reducing associated inflammation and inducing adhesion [133].

Lack of rapid diagnosis and prompt initiation of treatment is accompanied by a high death rate, with epidemiological data in TB-endemic countries showing that 46% of TB cases are undiagnosed at time of death [119]. The morbidity of these patients is also high, with 88% of deaths secondary to bacilli dissemination [140]. The statistics are not more favorable in the case of children either, tubercular pericarditis being found in 21–44% of children with TB, a percentage lower than the real prevalence due to underdiagnosis [141,142]. A recent study published by Watch et al. [143] highlights the high death rate of about 25% among children with TB pericarditis, highlighting the fact that TB continues to be a public health problem in the face of advancing technology and industrialization [144].

### 4.4. Aortic Involvement

Tuberculous aortitis is an extrapulmonary form of TB rarely encountered in clinical practice (0.3% of total cases with TB), but which poses diagnostic and treatment challenges alike [24,145]. The average time interval from the onset of symptoms to the start of anti-tuberculosis treatment is 18 months.

First mentioned in the literature in 1882, these cases have an incidence of less than 1% and are often a consequence of the spread of bacilli at a distance [146]. Aneurysms located in the descending thoracic or abdominal aorta (about 50% of cases) are the most common findings in patients with these extrapulmonary forms of TB [147]. Infrequently, perforation of adjacent structures may occur, which can lead to death in the absence of rapid diagnosis [148,149]. Involvement of the ascending aorta or aortic arch are extremely rarely reported to date [150,151,152].

There are cases in which MTB has been confined to the aortic valve, with the appearance of the inflammatory process at this level and causing aortic stenosis over time [153]. Histopathological identification of inflammatory cells, multinucleated giant cells, and fibrotic lesions guide this diagnosis [154]. Numerous MTB and *M. bovis* genes have been identified in the aortic wall of patients with Takayasu arteritis, suggesting the role of these bacilli in modulating the immunopathogenic mechanisms of this pathology [155,156]. Tuberculous aortitis has also been reported in patients with HIV infection [157].

Although few cases have been reported in the literature to date, several pathophysiological mechanisms have been proposed, the most commonly stated being direct dissemination from tuberculous lymph nodes, hematological dissemination via intima, or septic embolization of the arterial wall via vasa vasorum or lymphatics [148,158,159]. Tubercular infection of the aortic wall causes necrosis and subsequent rupture of the aortic wall, with the appearance of a major hemorrhagic episode or the formation of a perivascular hematoma communicating with the lumen of the aneurysm [160].

Symptoms of patients with tuberculous aortitis can range from classic systemic TB infection to dysphagia or hoarseness secondary to aneurysm-associated mass effect [161,162]. In a patient with fever and atherosclerotic lesions it is recommended to consider tuberculous aortitis in the differential diagnosis algorithm [163]. The presence of gastrointestinal bleeding episodes as the first symptom may occur in tuberculous aortitis complicated with aortoduodenal fistula [164]. Patients with tuberculous aortitis have a significant inflammatory process affecting other organs, causing secondary inflammatory processes such as pleuritis or spondylitis [165].

The therapeutic management of these cases often requires analysis within a multidisciplinary team [166]. The administration of antituberculosis medication and surgery are the main therapeutic tools applicable to these patients, with a high death rate of up to 50% [149,167,168]. The first reconstruction of a tuberculous aortic aneurysm using an artificial graft took place in 1955 [169]. In particular situations it is recommended to continue drug treatment for more than one year in order to prevent reinfection of prostheses or anastomosis sites [170].

### 4.5. Intracardiac Tuberculoma and Papillary Muscle Enlargement

Intracardiac tuberculoma and papillary muscle enlargement are cardiovascular complications rarely seen in clinical practice and are often autopsy findings [34,171]. There are extremely few cases presented in the literature in which papillary muscle enlargement is observed by using cardiac magnetic resonance imaging, the one presented by Das et al. [34] being one of them.

Intracavitary tuberculomas are predominantly located in the right heart, on the right atrial wall [172,173]. Their characterization differs; they may be single or multiple [174] and may result in obstruction of the right ventricular, superior vena cava, or coronary artery ejection tracts [175]. In some cases these formations may have functional consequences in the form of ventricular dysfunction, ventricular rupture, aortic regurgitation, or cardiac arrhythmias [34,176,177]. A recently published clinical study shows a link between myocardial nodules and the development of ventricular aneurysms, but further clinical studies are needed to establish pathophysiological connections [178].

The management of these cases is often difficult, requiring a multidisciplinary approach to identify optimal therapeutic solutions for each individual patient. Nuclear magnetic resonance allows the analysis of intracavitary structures, the tuberculous formations having a characteristic aspect on T2 images represented by the isointense central caseum, a hypointense area specific to the fibrous capsule, and the hyperintense line representative of the infiltrative inflammatory cell layer [34,55].

The diagnosis of certainty is established by seeding the infected tissue fragment on special culture media. Current clinical guidelines do not specify a therapeutic strategy indicating whether drug treatment is sufficient or surgery is necessary [179,180]. Some clinical studies present as a therapeutic model the administration of antituberculosis treatment for 6 months and subsequent surgical treatment in case of lack of symptom relief or complications [179]. Hashmani et al. [180] also reported the disappearance of an intracavitary mass in the right ventricle one year after starting TB-specific infectious drug treatment.

### 4.6. Cardiotoxic Effect of Antituberculosis Medication

In addition to the mechanisms associated with myocardial injury in TB described above, the cardiotoxic effect of antituberculosis medication (as moxifloxacin, bedaquiline, and delamanid) should not be neglected. Several data in the literature describe damage to the excito-conductive system, with the possibility of QT interval prolongation, although without mentioning severe consequences. It is worth mentioning that recent results show that the combination of bedaquiline and delamanid—the first drugs belonging to new therapeutic classes approved for the treatment of TB in the last decades—does not show significant QT prolongation compared to monotherapy [181]. Even so, in the context of current polymedicine, the association of other drugs with potential QT prolongation may lead to severe consequences.

It has recently been proposed to use an algorithm to assess the cardiac drug risk. It uses a decision tree, taking into account the medication administered and the iatrogenic risk as well as ECG changes other than those related to the QT interval. An adequate extrapolation from in vitro to in vivo results has been observed, thus opening up the prospect of using such an algorithm in the early development phases of new antituberculosis drugs [182].

Given the varied cardiac impairment that occurs in TB patients, the high degree of drug resistance, and the potential side effects associated with medication, we reiterate the importance of awareness of cardiac impairment, with multiple economic and medical implications alike. Also, the diagnosis of TB requires a cardiological evaluation to identify an associated extra-pulmonary form.

## 5. Conclusions

TB continues to be a global public health problem. Severe, multi-drug resistant forms, with difficulties in therapeutic management, are a worrying reality. The development of new anti-tuberculosis drugs to treat these forms has been difficult, with the first new agents emerging more than four decades apart. Moreover, cardiovascular damage significantly worsens the patient’s functional and vital prognosis. The association of pericarditis in all its forms and the possibility of the development of constrictive pericarditis, the occurrence of concomitant myocarditis with severe systolic dysfunction and complication with acute heart failure phenomena, and the long-term development of aortic aneurysms with risk of complications, as well as drug-induced toxicity, pose complex additional problems in the management of the TB patient. In this regard, a multidisciplinary cardiologist–pneumonologist–infectious disease specialist approach combined with modern means of risk estimation may be the way to effectively manage these patients.
